# Tuberculosis and pulmonary candidiasis co-infection present in a previously healthy patient 

**Published:** 2016-06-30

**Authors:** Dilia Mildret Fontalvo, Gustavo Jiménez Borré, Doris Gómez Camargo, Neylor Chalavé Jiménez, Javier Bellido Rodríguez, Bernarda Cuadrado Cano, Shirley Navarro Gómez

**Affiliations:** 1 Departamento de Postgrado, Doctorado en Medicina Tropical, Universidad de Cartagena, Cartagena, Colombia.; 2 Grupo de investigación UNIMOL. Universidad de Cartagena, Cartagena, Colombia; 3 Unidad de Cuidados Intensivo Adultos, Departamento de Medicina Interna, Clinica Nuestra. Cartagena, Colombia

**Keywords:** Invasive candidiasis, pulmonary tuberculosis, coinfection, immunocompetent, pulmonary fungal infection

## Abstract

**Background:**

The coexistance among fungal pathogens and tuberculosis pulmonary is a clinical condition that generally occurs in immunosuppressive patients, however, immunocompetent patients may have this condition less frequently.

**Objective:**

We report the case of an immunocompetent patient diagnosed with coinfection *Mycobacterium tuberculosis *and *Candida albicans*.

**Case Description:**

A female patient, who is a 22-years old, with fever and a new onset of hemoptysis.

**Clinical findings and diagnosis:**

Diminished vesicular breath sounds in the apical region and basal crackling rales in the left lung base were found in the physical examination. Microbiological tests include: chest radiography and CAT scan pictograms in high resolution, Ziehl-Neelsen stain, growth medium for fungus and mycobacteria through Sabouraudís agar method with D-glucose. Medical examinations showed *Candida albicans *fungus and* Mycobacterium tuberculosis *present in the patient.

**Treatment and Outcome:**

Patient was treated with anti-tuberculosis and anti-fungal medications, which produced good responses.

**Clinical relevance:**

Pulmonary tuberculosis and fungal co-infection are not common in immunocompetent patients. However, we can suspect that there is a presence of these diseases by detecting new onset of hemoptysis in patients.

## Abstract (es)

**Antecedentes:**

La coexistencia entre los hongos patógenos y la tuberculosis pulmonar es una condición clínica que se produce generalmente en pacientes inmunosuprimidos, sin embargo, los pacientes inmunocompetentes puede tener esta condición con menor frecuencia.

**Objetivo:**

Presentamos el caso de un paciente inmunocompetente con diagnóstico de una coinfección de tuberculosis *Mycobacterium tuberculosis *y *Candida albicans*.

**Caso clínico:**

Paciente femenina de 22 años con cuadro abrupto de tos, fiebre y hemoptisis sin antecedentes de enfermedad.

**Hallazgos clínicos y métodos diagnósticos:**

Al examen respiratorio se halló disminución del murmullo vesicular en la región apical y estertores crepitantes basales en el pulmón izquierdo. Se realizó estudios microbiológicos de muestras tomadas por expectoración y por fibrobroncoscopia en el que se incluyó la tinción de Ziehl-Neelsen, cultivo para micobacteria y hongos en medio Agar Dextrosa Sabouraud y filamentización en suero obteniéndose positividad para *Mycobacterium tuberculosis *y *Candida albicans*.

**Tratamiento y resultado:**

Se le realizó manejo con antifímicos de primera categoría y antimicóticos con buena respuesta clínica.

**Relevancia clínica:**

La coinfección fúngica y tuberculosis pulmonar no es frecuente en pacientes inmunocompetentes, debe sospecharse en episodios abruptos de hemoptisis.

## Introduction

 Tuberculosis (TB), which is an infectious disease, is still a serious threat for the population, despite all the efforts taken so far in this regard in the whole world. According to the World Health Organization, 9.6 million people (Range: 9.1-10 million) were detected presenting new cases of tuberculosis, and 1.5 million deaths (Range: 1.1-1.7 million) associated with this disease in 2014, from which 1.2 million Tuberculosis cases (12%) were related to HIV infections 1. In 2011, about 11,708 TB cases were detected in Colombia, from which 10,731 were new cases, while the others were cases previously treated (572 relapses, 97 failures, 308 abandoned cases that were retaken) 2. 

 There are many different types of pulmonary and extra-pulmonary diseases related to TB, which might have serious problems in differential and therapeutical diagnosis. These cases might also be aggravating factors associated with chronic obstructive pulmonary diseases, diabetes, elderly patients, high cholesterol levels, lung cancer, immunodeficiency, and fungal lung infections. In recent times, there has been a strong interest in fungal infection diagnosis due to the fact that patients presenting fungal diseases have presented several lung infections such as TB. The frequency of these infections and the number of immunosuppressive disease cases have gradually increased; hence it is necessary that medical personnel conduct clinical research in potentially pathogenic secondary fungal infections, considering that it can affect the progression of this disease, which can be lethal 3,4.

 Our aim is to report the case of an immunocompetent patient diagnosed with *Mycobacterium tuberculosis *and *Candida albicans* coinfections.

##  Clinic case

 A 22 years old mixed-race female patient, showed up into the Urgency room in a second level hospital in Cartagena de Indias (Colombia) with a clinic profile with six days of 38° C Intermittent fever and persistent cough. She worked as a receptionist and denies epidemiologic contact with suspicious people or the presentation of massive Hemoptysis in the fifth day of fever episode. There were no signs of comorbidity deceases, found; neither: smoking, medicament consume, psychoactive substances consumption nor Tuberculosis diagnosis. She has not been outside the city where she is living lately. In the family history files were found Hypertension and non-classified Hyperlipidemia.

 In the physical examination was found a breathing frequency of 32 respiration/ per min, a heart rate of 84 beats/ min, a temperature of 38° C, the arterial pressure was in 110/80 mmHg and finally, a weight of 51kg, a height of 1.64 m and a CMI of 19.

 The patient presented a good muscular state. It presented general pallor, plus a pale and wet mucosa with a good perfusion. In the clinical revision of the skin, it was not found any active nor former infection in the mucosa or the oral cavity. In the respiratory exam, it was found a decrease in vesicular murmur in the apical region and basal crackling rales in the left lung. The rest of the physical exam showed normal results. The complete blood count showed the hemoglobin in 10.7 g/dL; The mean corpuscular volume (MCV): 103 µm^3 ^; Red blood cell distribution width (RDW): 14.8% (normal rate: 12%-15%), Leukocytes: 8,400/mm^3 ^(60% Neutrophils, 27% Lymphocytes, 8% Monocytes, 5% Eosinophils) and the platelets: 262,000/mm^3^.

**Figure 1.**
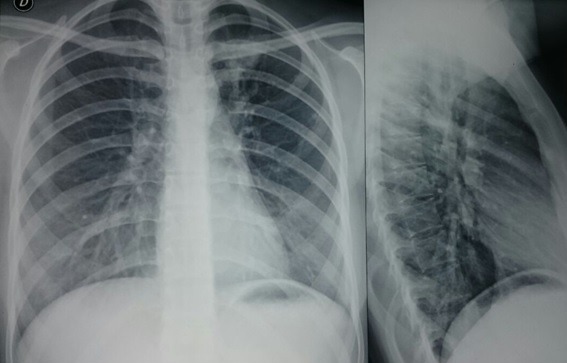
Front to back and lateral chest x-rays. It was taken in the fifth day after the symptoms started, where it's identified a revetment in the soft parts of the normal thorax. The diaphragm is in the normal position and free Costophrenic angles. It is appreciated the increase in density of the interstitial left apical location with interstitial left apical in relation to the regarding consolidative process. The pulmonary vasculature or the aorta showed no alterations. The situation and the limits of the trachea are normal. No paratracheal lines widening and no displacement of the different mediastinal lines.

 The arterial gases, serum electrolytes, gel electrophoresis of proteins, coagulation tests, quantification of serum immunoglobulins, renal (partial urine and urinary sediment, creatinine, and ureic nitrogen) and hepatic assessment studies (bilirubins, alanine transaminase, aspartate transaminase, alkaline phosphatase, serum albumin, prothrombin time) went normal. The ELISA test for HIV went negative. The Anti-nuclear antibodies and bacterial Anti-DNA resulted negative. The purified protein derivative (PPD) was 7 mm after 72 h of the test. The imaging studies informed in the chest radiograph an area of increase in density of the interstitial left apical location with interstitial left apical in relation to the regarding consolidative process (Fig. 1). 

**Figure 2.**
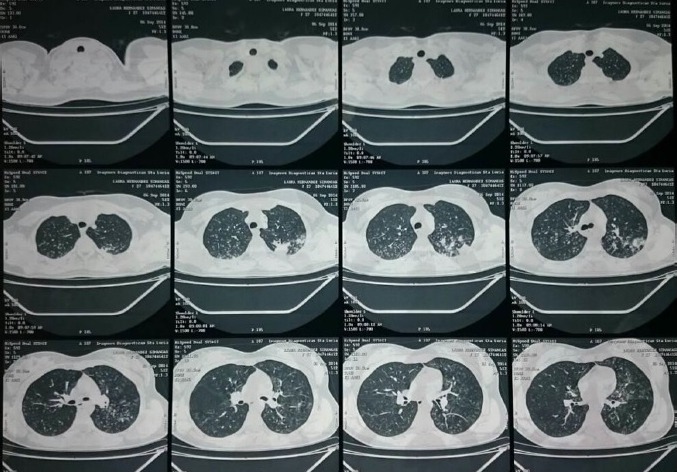
Torax CAT in hight resolution. The soft tissues and the osseous structures of the thoracic walls show no alterations. There is no evidence of pleural effusions nor intrapleural or extrapleural lesions. It is observed a thickening and an alveolar occupation with cavitation apical posterior left upper lobe area finding nodules with budding tree pattern in both lung apexes. The trachea, the Mainstem bronchi and lobar bronchi which are visualized are normal. There is no evidence of mediastinal masses. The cardiac silhouette, big blood vessels and other vascular structures visualized do no show pathologic change significance.

 In the computerized axial tomography (CAT) scan of the thorax it was observed a thickening and an alveolar occupation with cavitation apical posterior left upper lobe area. It was taken a high resolution CAT scan of the thorax finding nodules with budding tree pattern in both lung apexes. (Fig. 2).

 A fiberbronchoscopy and a bronchoalveolar Lavage was taken, where it was visualized, in the left lung upper lobe region, the erythematous and edematous endobronchial mucosa with yellowish-white nodules plus necrotic areas and cavitary lesion with serohematic secretion, which was taken from a sample in a protected catheter. The sample was analyzed after an hour and 17 min, with fresh results from the study and the Gram staining that showed a branching structure of budding yeast and a negative Ziehl-Neelsen stain. The culture in the Ogawa-Kudoh showed positive to *M. tuberculosis. *We obtained the growing of more than 30 *C. albicans *colonies with pseudomiceliales forms for fungus cultivation in Sabouraud agar medium (SDA) and positivity to triplicate serum filamentization . The same studies were done in a spontaneous hemoptoic sputum simple, obtaining the same results. The sample was paired in the laboratory of the National Institute of Health in Colombia with cultivation in a liquid media by MGIT (Mycobacterial Growth Indicator Tube)-960 with a growth of *M. Tuberculosis *sensitive to medicinal products from the category I (isoniazid, rifampicin, pyrazinamide, and ethambutol). It was handled with voriconazol for the infection by *Candida *Directly Observed Treatment, Short Course (DOTS) for tuberculosis with isoniazid (300 mg/day), rifampicin (600 mg/day), pyrazinamide (1,600 mg/day) and ethambutol (1,100 mg/day) for 48 doses, and continuing the second phase with isoniazid and rifampicin 150 mg/day three times a week each for 54 doses. The patient showed an improvement in the symptoms after the beginning of the treatment and was released from the hospital after 15 days.

## Discussion

 The frequency of opportunistic mycotic infections presence grows in a progressive way due to an increase in the number of immunosuppressive diseases. *Candida albicans *is still the most common fungus associated with infections in immunocompromised patients 5,6. The prevalence of pulmonary tuberculosis co-infection cases with *Candida *is about 15-32% in different studies 7,8. The most common species of these fungus (from 9 to 80%) is the *C. albicans, *which is a mucous membrane found as a component of the habitual normal microflora in the digestive tract. Also, this fungus is considered ubiquitous in a hospital environment, whether it is in the air, and/or in inert surfaces such as floors and roofs and in the food as well 8.

*Candida albicans* has evolved as a potentially pathogenic fungus, instead of an innocuous guest in mucous material of patients presenting with bronchopulmonary diseases, which might increase the complications in these diseases. Kali *et al.* 5, found that 40% of Candida *c*o-infection cases presented* C. albicans *(59%), followed by *C. tropicalis *(20%) and *C. glabatra *(20%). However, many authors have determined *Candida* species as the most common fungal agent isolated from patients' mucus production present in pulmonary tuberculosis cases. The study about the importance of these infections has always been a controversial matter due to the fact that almost 32.5% of healthy people are carriers of *Candida *in their throat 9. This situation might lead to the contamination of the sputum sample. In order to eliminate this problem, many different operational strategies have been used while taking microbiological samples in the lesions of lung parenchyma. In the clinical case of our patient, samples were obtained through flexible bronchoscopy, and the* Candida* infection diagnosis was made because of the presence of fungal budding found in the smear, taking into account Kahanpaa's criteria 10. According to Kahanpaa, the isolation of 3 or more colonies of *Candida*, from more than 30 colonies with pseudomycelia organisms in the SDA medium, to be an infection rather than a colonization 11.

 During a study of patients with comorbidities presenting Tuberculosis diagnosis in the city of Porto Alegre, Unis *et al*. 12, found a prevalent fungal infection related to *Aspergillus fumigatus* (57%), followed by *A. niger* (29%), *Scedosporium apiospermum *(7%) and *A. flavus *(7%). All the patients presented hemoptysis, others clinical manifestations such as cough, weight loss, fever, dyspnea, purulent sputum, asthenia and breast pain.

 The studies of the patients' image presenting TB were with normal x-rays in15%, with a suggestion of making studies as pulmonary CAT. In addition, CAT high resolution is used to determinate the active disease, in which is possible to see images with linear structures of multiple ramifications with similar caliber from an unique "steam" ("budding tree" aspect), which is common in patients presenting with a wide bronchogenic diffusion 13,14.

 Our patient does not have medical history nor clinical data explaining a state of immunosuppression situation, Tuberculosis and pulmonary Candidiasis co-infection. The new onset of hemoptysis of the patient, previously healthy, led to her hospitalization so that we could make breath and general studies in order to find underlying diseases explaining the mycotic and fungal co-infection.

 The pathologic change in the respiratory tissue, produced in patients with TB, contributes to the favorability in their pathogenesis of the mycotic infection, which must be detected in patients with TB severe symptoms such as new onset of hemoptysis. 

## Conclussion

 Tuberculosis co-infection and mycotic infection are not common in patients without evidence of comorbidities or immunosuppressive diseases. Even though the synergistic growth between *Candida* and *M. tuberculosis *is well described, the isolated respiratory specimens are usually ignored considering them as pathogenic commensal. The suspicious diagnostic must include patients presenting massive hemoptysis or with an unsuitable response to antifimic treatments. This findings will allow researchers to make studies concerning to pathologies and situations involving immunosuppression. 
